# Bio-Inspired Photosynthesis Platform for Enhanced NADH Conversion and L-Glutamate Synthesis

**DOI:** 10.3390/polym16152198

**Published:** 2024-08-01

**Authors:** Junxiao Tang, Zhenyu Liu, Rongjie Wang, Yanze Wang, Zhaoyong Zou, Jingjing Xie, Pengchao Zhang, Zhengyi Fu

**Affiliations:** 1 State Key Laboratory of Advanced Technology for Materials Synthesis and Processing, Wuhan University of Technology, Wuhan 430070, China; tangjunxiao@whut.edu.cn (J.T.); rjwang@whut.edu.cn (R.W.); 290694@whut.edu.cn (Y.W.); zzou@whut.edu.cn (Z.Z.); 2 International School of Materials Science and Engineering, Wuhan University of Technology, Wuhan 430070, China; 322092@whut.edu.cn

**Keywords:** layered vaterite, self-assembly, biocatalysis, photocatalysis, artificial photosynthesis

## Abstract

Inspired by the layered structure, light absorption, and charge carrier pathway of chloroplast thylakoids in natural photosynthesis, we propose a novel artificial photosynthesis platform, which is composed of layered structured vaterite as the scaffold with gold nanoparticles (AuNPs), photosensitizer eosin Y (EY), and redox enzyme L-glutamate dehydrogenase (GDH) as the functional components. The EY exhibited significantly enhanced light absorption and charge carrier generation due to the localized surface plasmon resonance (LSPR) around the AuNPs and light refraction within the layers. This artificial photosynthesis platform can regenerate reduced nicotinamide adenine dinucleotide (NADH) under visible light and promote the rapid conversion of α-ketoglutarate to L-glutamate (0.453 Mm/h). The excellent biocompatibility of layered vaterite significantly enhances the resistance of GDH to harsh conditions, including high pH (pH = 10) and elevated temperatures (37–57 °C).

## 1. Introduction

Studying the process of photosynthesis is promising for converting solar energy to renewable energy [1,2]. Plants use chloroplast pigments to capture photon energy and generate electrons, which are guided to catalytic centers to convert NAD^+^ to NADH, enabling the Calvin cycle [3,4]. The precise arrangement of nanostructures in leaves serves as a mechanical support for them [5]. Various methods have been employed to develop efficient and sustainable artificial photosynthesis platforms [6,7,8]. The distance between functional components is crucial in colloid systems [9]. A common artificial photosynthesis platform is assembled from polydimethylsiloxane (PDMS) and polymethyl methacrylate (PMMA). Changes in the location and structure of polymers can affect the distance between photosensitizers and catalysts, thereby impacting charge transfer within the system [10,11]. Therefore, a structurally stable and controllable microenvironment is required to coordinate complex electrochemical reactions.

To date, considerable efforts have been made to develop an integrated platform for immobilizing the light-capturing components of photosynthesis [12,13]. A promising approach involves incorporating these photosynthetic active components into a 3D scaffold matrix resembling thylakoid structures and maintaining their unique functionalities for use in the regeneration of NADH and the conversion of α-ketoglutarate into L-glutamate. Such platforms include microfluidic chips [9], peptide nanotubes [14], and P22 virus-like particles [15]. However, the preparation of PDMS for microfluidic chips, which requires silicon molds made by soft lithography and precise operations, is expensive and complex, and the enclosed spaces have weak recyclability. In peptide nanotubes and P22 virus-like particles platforms, the photocatalysts and redox enzymes are exposed in solution and have poor tolerance to harsh conditions such as high temperatures and high pH.

Solving the aforementioned issues is crucial for the development of photosynthesis platforms. In fact, calcium carbonate (CaCO_3_), the most common biomineral in nature, has been used as a carrier for various proteins or drugs to cope with harsh conditions. Its preparation is simple, cost-effective, structurally controllable, and biocompatible, making it a promising platform for integrating photosynthesis [16,17]. It exists in three crystalline phases: calcite, aragonite, and vaterite [18]. Among these, vaterite exhibits the highest porosity and surface area, making it a promising material [19]. Various morphologies of vaterite have been prepared, including spherical [20], lamellar [21], and flower-like morphologies [22]. If vaterite not only retains its porous structure but also exhibits layered characteristics akin to thylakoid membranes [23], this would provide it with a significant surface area and light refraction advantages, leading to higher efficiency in light energy conversion. The biocompatibility and large surface area of vaterite provide sufficient space for the light-capturing components of photosynthesis to withstand harsh external conditions. Moreover, the outstanding recyclability of CaCO_3_ plays a significant role in promoting sustainability [24]. AuNPs exhibit excellent electrical conductivity and catalytic performance [25,26]. Additionally, AuNPs can further enhance light absorption through surface plasmon resonance, effectively exciting the photosensitizer [27]. Depositing AuNPs on vaterite contributes to enhancing the effective capture of solar energy.

In this study, inspired by the photoconversion function of thylakoids in chloroplasts during natural photosynthesis, we developed a thylakoid-like vaterite with a layered structure, employing it as a 3D scaffold to encapsulate the photosensitizer EY and GDH. These functional components facilitate light absorption and charge carrier generation, constructing a bio-inspired photosynthesis platform. Taking advantage of the light-refracting effect of layered vaterite structure and the enhanced light absorption of EY from the LSPR of AuNPs, solar energy is effectively converted into chemical energy, facilitating the regeneration of NADH and the conversion of α-ketoglutarate into L-glutamate. The protection provided by vaterite significantly enhances the tolerance of GDH to harsh conditions such as high pH (pH = 10) and high temperature (37–57 °C).

## 2. Materials and Methods

### 2.1. Materials

Calcium chloride anhydrous (99.99% CaCl_2_), ammonium carbonate (99% CH_8_N_2_O_3_), ammonium sulfate (99% H_8_N_2_O_4_S), sodium borohydride (98% NaBH_4_), gold chloride hydrate (Au ≥ 47.5% HAuCl_4_), methanol (99.9% CH_4_O), ethanol (99.9% C_2_H_6_O), triethanolamine (TEOA, 99% C_6_H_15_NO_3_), rhodamine B (RhB, 98% C_28_H_31_ClN_2_O_3_), eosin y (EY, 99% C_20_H_6_Br_4_Na_2_O_5_), α-ketoglutaric acid (98% C_5_H_6_O_5_), β-nicotinamide adenine dinucleotide (NAD^+^, 98% C_21_H_27_N_7_O_14_P_2_), hexadecyltrimethylammonium bromide (CTAB, 99% C_19_H_42_BrN), l-glutamic dehydrogenase from bovine liver, 10× phosphate-buffered saline (PBS).

### 2.2. Preparation of Layered Vaterite and Deposition of AuNPs

Preparation of AuNPs: In a typical procedure, a solution of 70 mM CTAB and 0.1 M HAuCl_4_ was stirred for 5 min. Subsequently, 0.6 mM NaBH_4_ was slowly added, and the pH was adjusted to 11 with NaOH. The color of the solution gradually changed from pale yellow to wine red. Afterward, the sample was centrifuged 3 times at 12,000 rpm and redispersed in deionized water. The synthesis of CaCO_3_ was carried out based on the gas diffusion method. First, a 40 mM CaCl_2_ solution was prepared under stirring and a certain amount of RhB and CH_3_OH was added. Next, 3 g of finely powdered (NH_4_)_2_CO_3_ was placed into a culture dish with a diameter of 60 mm (φ 60 mm). A larger culture dish with φ 150 mm was utilized as the sealed system for the growth of CaCO_3_. The dish containing the CaCl_2_ solution was irradiated with a 350 W xenon lamp. The product was washed with deionized water and ethanol, then dried and stored under vacuum. The deposition of Au involved dispersing 20 mg of CaCO_3_ into 10 mL of ethanol solution. The volume ratios of CaCO_3_ to Au were 4:1, 2:1, and 1:1, respectively, labeled as vaterite-Au1, vaterite-Au2, and vaterite-Au3. After being placed in a rotating mixer for 30 min, the samples were collected, centrifuged, washed, and dried before being stored in a vacuum oven.

### 2.3. Preparation of [M]^+^

A total of 123.6 mg of dichloro (η^5^-pentamethylcyclopentadienyl)rhodium (III) dimer ([CpRhCl_2_]_2_) was dissolved in 10 mL of methanol, forming a red suspension. Subsequently, 62.4 mg of 2,2-bipyridine was added, leading to the formation of an orange solution. The solution was then concentrated under vacuum to a final volume of 5 mL. Ether was added dropwise to the concentrate at 4 °C to precipitate [CpRh(bpy)Cl]Cl, which was collected by vacuum drying. Finally, 40 mL of H_2_O was added to dissolve the precipitate and achieve a concentration of 10 mM of [M]^+^.

### 2.4. Characterization

The morphology of CaCO_3_ particles was observed using a field emission scanning electron microscope (FESEM, SU8020, Hitachi, Tokyo, Japan) under an accelerating voltage of 5 kV. Au-CaCO_3_ was characterized using a transmission electron microscope (TEM) (JEM-1400plus, JEOL, Tokyo, Japan) operating at 200 kV voltage coupled with selected area electron diffraction (SAED) and scanning transmission electron microscopy (STEM) images (Talos F200S, Thermo Scientific, Hillsboro, OR, USA). Photoluminescence spectroscopy (PL) (FLS980 spectrometer, Edinburgh Instruments, Livingston, UK) was employed with 450 nm light excitation to evaluate the separation efficiency of electrons and holes in the samples. Fluorescence microscopy images were obtained using an observer 7 inverted fluorescence microscope (Zeiss, Oberkochen, Germany). The pore size and surface area of CaCO_3_ were measured using the Brunauer–Emmett–Teller (BET) method with a fully automatic surface area and pore size analyzer (Micromeritics ASAP 2460, Norcross, GA, USA). The zeta-potential of CaCO_3_ suspended in deionized water (10 mg mL^–1^) at 25 °C was measured using a zeta-potential analyzer (Zetasizer ultra, Malvern, Worcestershire, UK), repeated three times. UV–visible diffuse reflectance spectroscopy (UV-Vis, UV-2550) and steady-state/transient fluorescence spectroscopy (Edinburgh FLS1000, Livingston, UK) were used to characterize the photocatalytic performance. The voltametric experiment was conducted utilizing a three-electrode system comprising a glassy carbon disk as the working electrode, a platinum wire as the counter electrode, and an Ag/AgCl electrode serving as the reference electrode assembly. Electrodes were interfaced with a potentiostat (CHI760E, Shanghai, China). The electrodes were coated with a solution of dodecyltrimethylammonium bromide (DDAB) (10 mM, 5 μL) and air-dried at room temperature. Subsequently, 20 μL of vaterite-Au-EY (10 *w*/*v* %) solution was drop-cast onto the glassy carbon disk and allowed to dry overnight. The buffer solution used was phosphate buffer (10 mM, pH 8). A xenon lamp was employed as the light source with a light–dark cycle of 20 s (light on for 20 s, off for 20 s), repeated for more than four cycles. The initial potential (E) was set at 0.5 V, with a sampling interval of 0.1 s, a run time of 250 s, and a sensitivity of 1 × 10^−6^ A/V.

### 2.5. NADH Conversion and L-Glutamate Synthesis

The reaction solution consisted of 1 mM NAD^+^, 0.25 mM [M]^+^, and 0.1 M TEOA dissolved in a phosphate-buffered solution (0.01 M, pH 8), including dispersed vaterite-Au-EY. The photochemical regeneration of NADH was conducted using an LED with a power of 10 mW/cm^2^. Absorbance was measured using UV-Vis spectroscopy. The concentration of NADH was calculated by measuring the absorbance at 340 nm. For the photo enzymatic conversion reaction, the 3 ml phosphate buffer (0.01 M, pH 8) solution comprised 50 mg vaterite-Au-EY, 0.1 M TEOA, 1 mM NAD^+^, 0.25 mM [M], 0.4 g/L α-ketoglutarate, and 0.1 M (NH_4_)_2_SO_4_. L-glutamate concentration was measured using ultra-high-performance liquid chromatography (UHPLC, agilent technologies 1290 infinity, Agilent Technologies, Santa Clara, CA, USA). The chromatographic column used was a C18 column with a mobile phase of 0.01% phosphoric acid, a flow rate of 1 mL/min, a detection wavelength of 203 nm, and a column temperature of 25 °C.

### 2.6. Stability and Reusability Tests

Thermal stability was tested by maintaining the reaction temperature between 27 °C and 57 °C using a vacuum drying oven. pH stability was assessed by altering the pH using the ammonia solution. The cycling test proceeded as follows: vaterite-Au-EY was employed for L-glutamate reduction for one hour, after which the samples were centrifuged, collected, and then redispersed in a fresh reaction medium.

## 3. Results and Discussion

### 3.1. Morphology and Structure of Vaterite-Au

**Figure 1:**
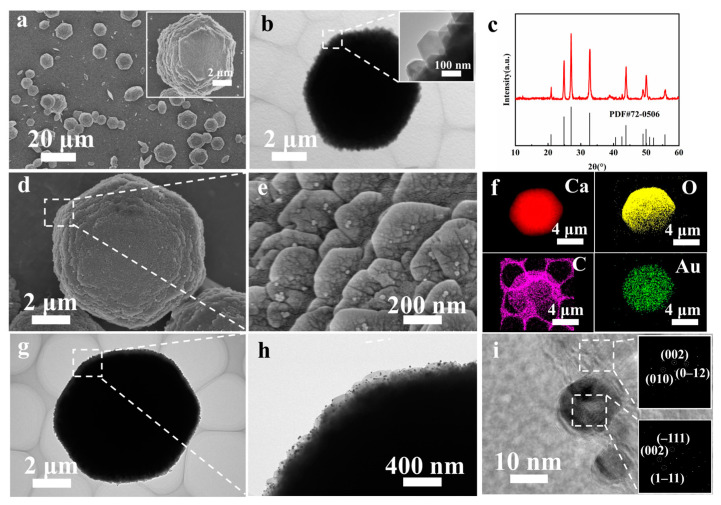
(**a**) SEM image of CaCO_3_, (**b**) TEM image of CaCO_3_, (**c**) XRD spectra of CaCO_3_, (**d**) SEM image of CaCO_3_ after deposition of AuNPs, (**e**) high-magnification SEM image, (**f**) elemental mapping, (**g**) TEM image of CaCO_3_ after deposition of AuNPs, (**h**) high-magnification TEM image, (**i**) high-resolution TEM image.

In chloroplasts, thylakoids possess a unique layered structure that facilitates light absorption and transfer. Moreover, the thylakoid structure provides protection and support for various enzymes involved in photosynthesis. Inspired by thylakoids, we have constructed a bio-inspired photosynthesis platform with similar functionalities. The SEM image in Figure 1a shows that CaCO_3_ prepared under the synergistic effect of RhB and light exhibits a layered morphology. The layered morphology is distinctly observed and composed of hexagonal sheets stacked together. The size of the layered CaCO_3_ ranges from 5 to 10 μm. TEM images (Figure 1b) show that the hexagonal structures have sharp angles and exhibit 120° angles, and the layered structure is self-assembled from smaller hexagonal sheets. XRD spectra (Figure 1c) indicate that pure-phase layered hexagonal vaterite can be synthesized under illumination. Figure 1d demonstrates that the deposition of AuNPs has no effect on the layered morphology of CaCO_3_. Figure 1e confirms the uniform distribution of AuNPs on the surface of layered CaCO_3_. Elemental mapping (Figure 1f) further validates the uniform distribution of AuNPs on CaCO_3_. The TEM images in Figure 1g illustrate that the layered CaCO_3_ nanoparticles exhibit a typical hexagonal morphology, with AuNPs uniformly deposited on the CaCO_3_ layers (Figure 1h). The high-resolution TEM image (Figure 1i) reveals distinct lattice fringes, and SAED confirms that the nanoparticles crystallize as AuNPs, and the crystalline phase of CaCO_3_ is vaterite (Figure 1i illustration). Overall, SEM and TEM images confirmed that AuNPs were successfully and uniformly dispersed within the layered vaterite morphology, with no impact on the vaterite morphology.

**Figure 2:**
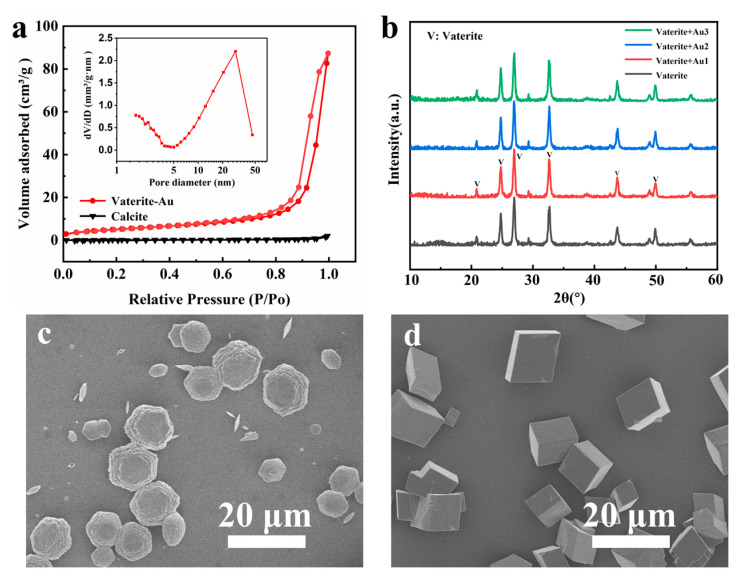
(**a**) Pore size distribution, (**b**) XRD spectra. SEM image of CaCO_3_ after 30 days at room temperature: (**c**) layered vaterite, (**d**) calcite.

BET analysis of the layered vaterite microspheres revealed an average pore size of 29.21 nm, pore volume of 0.13 cm^3^ g⁻^1^, and surface area of 18.49 m^2^ g⁻^1^ (Figure 2a). Compared to calcite (0.57 m^2^ g⁻^1^), the surface area increased 30 times. To investigate the effect of AuNPs concentration on the crystalline phase of CaCO_3_, XRD was conducted on CaCO_3_ loaded with varying concentrations of AuNPs. The XRD patterns (Figure 2b) showed sharp reflection peaks of vaterite, indicating that the deposition concentration of Au has no effect on the crystalline phase of CaCO_3_. Due to the nanoscale size of the Au particles and their relatively low concentration, the diffraction peaks became weak and broad, making it difficult to detect the diffraction peaks of Au in XRD. The morphological stability of the layered CaCO_3_ was also tested at room temperature for 30 days. SEM images show that the layered vaterite crystalline phase and morphology remained stable over 30 days (Figure 2c), while spherical vaterite had completely transformed into calcite (Figure 2d). This indicates that specific crystal faces of the vaterite were stabilized, hindering the dissolution and reprecipitation process required for the transformation into calcite, thereby reducing or preventing further growth or reorganization [28].

### 3.2. Composition and Structural of Vaterite-Au-EY

**Figure 3:**
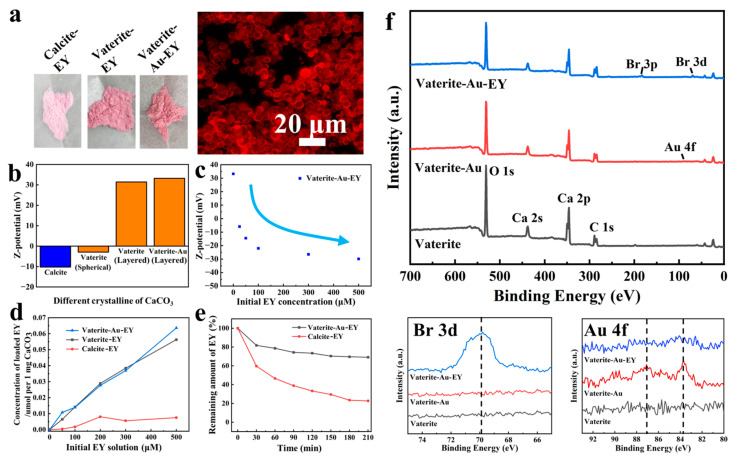
(**a**) Optical images and fluorescence microscopy images of vaterite-Au-EY, (**b**) zeta-potential of different crystalline of CaCO_3_, (**c**) zeta-potential of vaterite-Au-EY using different EY concentrations, (**d**) capacity of different CaCO_3_ loadings for EY, (**e**) retention capacity of EY on different CaCO_3_, (**f**) XPS spectra.

A composite hybrid with light-capturing capability was prepared by encapsulating the anionic dye EY as a photosensitizer within CaCO_3_. During the precipitation process, the EY dissolved in the reaction mixture was simultaneously captured by the electrostatic attraction within CaCO_3_. The vaterite-Au-EY and vaterite-EY composites exhibited a deep pink color, distinct from the lighter color of calcite-EY (Figure 3a), which indicates that vaterite is capable of capturing more EY. Fluorescence images revealed the distribution of EY within vaterite-Au-EY. Due to the complex surface charge of CaCO_3_, resulting from several ionic species (such as OH^−^, H⁺, CO_3_^2−^, Ca^2^⁺, and HCO_3_^−^) partially dissolving from its surface into the aqueous phase, the zeta-potential of calcite, spherical vaterite, layered vaterite, and layered vaterite after Au deposition were characterized (Figure 3b). Spherical vaterite commonly carries weak negative charges, while calcite exhibits more negative charges (−17 mV). Layered vaterite prepared with RhB under illumination displayed high positive charges due to the positive charge carried by the RhB, which facilitated the adsorption of anionic dye EY. Figure 3c shows that the zeta-potential of vaterite-Au-EY gradually decreases with the increase in EY content. This indicates that the positive surface of vaterite-Au promotes the electrostatic attraction between EY molecules, resulting in a gradual decrease in surface charge with the adsorption of EY. Additionally, we indirectly measured the concentration of loaded EY in vaterite-Au-EY by monitoring the change in EY concentration in the prepared solution after encapsulation. Figure 3d indicates that the amount of loaded EY is in the range of approximately 0.03 μmol/mg (200 μM). The EY loading in vaterite-Au was 10 times higher compared to calcite. This difference was attributed to the larger surface area of porous vaterite (18.49 m^2^/g) compared to calcite (0.57 m^2^/g). We further investigated whether the loaded EY leaked from vaterite-Au-EY and calcite-EY in deionized water. Over 80% of encapsulated EY molecules were retained within vaterite-Au-EY, while over half of the encapsulated EY was lost after 3 h of incubation when adsorbed on calcite (Figure 3e). This was attributed to the positive surface charge of layered vaterite, which favored the absorption of negatively charged EY. XPS analysis was utilized to further analyze the assembled powders of vaterite, vaterite-Au, and vaterite-Au-EY to confirm their composition (Figure 3f). All powders exhibited characteristic peaks originating from CaCO_3_ at Ca 2s, Ca 2p, C 1s, and O 1s [29]. The XPS spectrum of vaterite-Au clearly showed additional peaks at 83.8 and 87.2 eV binding energies corresponding to Au 4f [30], indicating the successful formation of AuNPs. The characteristic peaks of Br 3p at 190 eV and Br 3d at 69.6 eV in vaterite-Au-EY spectra confirmed the presence of EY in the samples [31]. Particularly, since XPS can only detect a few nanometers of the surface, the encapsulation by EY results in a decrease in the Au characteristic peaks. The notable reduction in the Au 4f characteristic peaks in the vaterite-Au-EY group compared to the vaterite-Au group indicates that most of the Au NPs are encapsulated by EY.

### 3.3. Photocatalytic Performance of the Vaterite-Au-EY Composite

**Figure 4:**
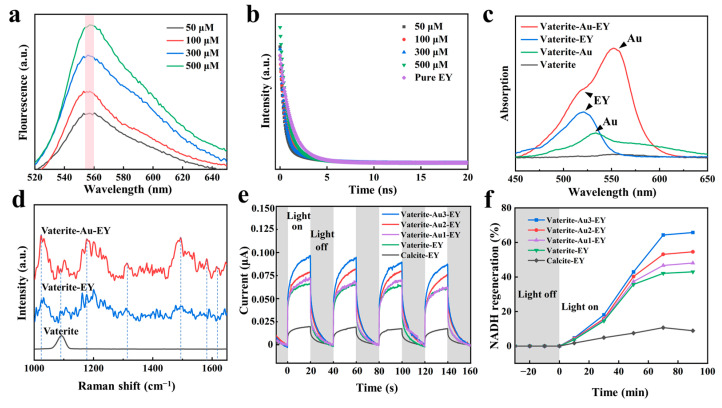
(**a**) PL spectra, (**b**) time-resolved fluorescence, (**c**) UV-Vis spectra, (**d**) Raman spectra, (**e**) amperometric current-versus-time (i-t) curve, (**f**) time curve of NADH photochemical regeneration.

We further investigated the photoelectronic properties of EY encapsulated on the surface of layered vaterite. As shown in Figure 4a, the fluorescence intensity of vaterite-Au-EY increases with the concentration of EY. We compared the fluorescence lifetimes of free EY and vaterite-Au-EY (Figure 4b). When measured using a biexponential function, the lifetime of vaterite-Au-EY was almost identical to that of free EY (1.37 ns). The LSPR of AuNPs and their interaction with EY were investigated using UV-Vis spectroscopy, with a comparison of the normalized absorption spectra for different mixed samples (Figure 4c). Compared to the vaterite group, the characteristic absorption peak of EY at 518 nm in vaterite-EY was easily identifiable [32]. For the vaterite-Au-EY group, the spectrum displayed two adjacent peaks due to the presence of both EY and AuNPs. Vaterite doped with AuNPs exhibited significantly enhanced absorption intensity at 518 nm compared to the vaterite-EY group. Additionally, a peak around 550 nm was observed, attributed to the LSPR of Au NPs [33], which shifted unevenly due to variations in the diameter and shape of the AuNPs. A red shift in the peak position was noted compared to bare AuNPs (532 nm) formed on the vaterite layer [34], as expected from the increased effective refractive index of the surrounding medium. This shift and the enhanced absorption intensity highlight the advantage of the layered vaterite structure, akin to thylakoid membranes, in optimizing light absorption and charge carrier generation. The strongest absorption occurred in the top EY layer, and the absorption intensity of EY in the vaterite-Au-EY group significantly increased compared to vaterite-EY. This enhanced light absorption of EY is attributed to the strong field enhancement generated by the LSPR of AuNPs. In vaterite-Au-EY, the EY molecules coating the Au core are located in the region of plasma field enhancement, leading to increased light absorption of EY due to the field distribution. Raman spectroscopy analysis further supports the field enhancement effect of the LSPR of AuNPs. Figure 4d presents the normalized Raman spectra of vaterite, vaterite-Au, and vaterite-Au-EY, obtained using 514 nm excitation. Compared to the vaterite-EY group, the Raman peaks of EY at 1620 cm^−1^, 1498 cm^−1^, 1339 cm^−1^, and 1180 cm^−1^ were significantly enhanced in the group containing AuNPs [35]. These peaks arise from typical vibrational modes in the pyridinium and benzene rings of EY. The peak at 1090 cm^−1^ corresponds to the carbonate ions in vaterite [36]. The amplified Raman signal demonstrates the enhanced electromagnetic field in vaterite-Au-EY resulting from the presence of AuNPs. After confirming that AuNPs enhance EY light absorption in vaterite, the capacity to regenerate NADH from NAD^+^ under visible light was investigated. This regeneration is a crucial step for performing photobiocatalytic reactions. According to the i-t curve in Figure 4e, the photocurrent of vaterite-Au-EY is amplified under illumination, exhibiting a much higher current density than calcite-EY. Additionally, the photocurrent density of vaterite-Au-EY increases with the concentration of Au. Subsequently, vaterite-Au-EY with [M], NAD^+^, and TEOA was employed for visible-light-driven NADH regeneration. After incubating with NAD^+^ (1 mM) for 1 h under irradiation, vaterite-Au-EY regenerated 65.8% NADH from NAD^+^, while Calcite-EY only exhibited an 8.9% conversion rate (Figure 4f). The significant increase in photochemical NADH regeneration efficiency is attributed to the difference in the amount of EY loaded in vaterite-Au-EY and calcite-EY. The regeneration rate of NADH is proportional to the Au concentration, supporting the notion that Au enhances the rapid transfer of photoexcited electrons and improves the light-harvesting reaction.

### 3.4. Biocatalytic Performance and Stability of Vaterite-Au-EY Composite

**Figure 5:**
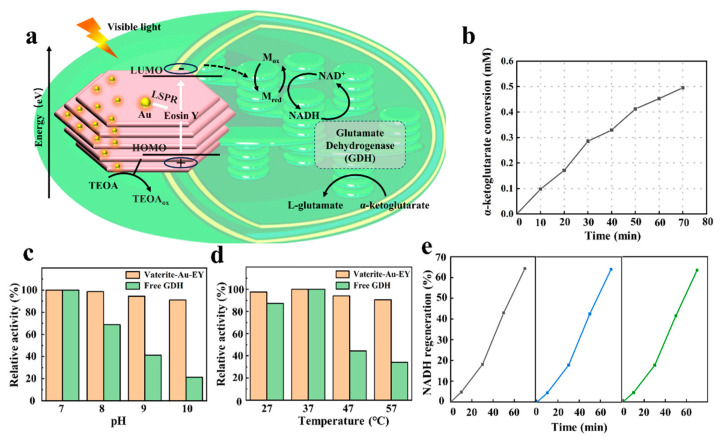
(**a**) Schematic diagram of the integrated biocatalytic artificial photosynthesis platform within layered vaterite, (**b**) α-ketoglutarate conversion, (**c**) relative activity under different pH, (**d**) relative activity under different temperatures, (**e**) activity of vaterite-Au-EY during repeated use.

Layered porous vaterite, with its high loading capacity and excellent biocompatibility, serves as an outstanding carrier for protein loading [37]. Vaterite-Au-EY is used as a scaffold, GDH is loaded into the layered vaterite, providing effective protection for the GDH [38]. As shown in Figure 5a, during the artificial photosynthesis process with vaterite-Au-EY, photoexcited electrons in EY are transferred to the electron mediator [M], facilitating the photochemical regeneration of NADH under visible light. Induced the catalytic conversion of α-ketoglutarate to L-glutamate by GDH. The visible-light-driven biocatalytic reaction in vaterite-Au-EY was evaluated by irradiating it with a white LED (power: 10 mW/cm^2^) for 70 min. As shown in Figure 5b, during the illumination period, L-glutamate is gradually formed from α-ketoglutarate, and the conversion rate remains stable, with an L-glutamate conversion of approximately 0.453 mM within 1 h. To highlight the advantages of layered porous vaterite as a scaffold for biocatalytic conversions, the effects of solution pH, reaction temperature, and recyclability on the light-driven biocatalytic conversions were investigated. Layered vaterite, as a carrier for GDH, provides protection against external factors. It has been reported that GDH loses activity at temperatures above 40 °C and high pH values [39,40]. Figure 5c shows the strong adaptability of GDH-loaded vaterite to highly alkaline environments. Compared to free GDH, vaterite-Au-EY maintains a high relative conversion rate at pH levels ranging from 7 to 10, significantly extending the adaptability of GDH to harsh pH conditions. Figure 5d demonstrates that layered vaterite also significantly enhances the thermal stability of GDH. At 57 °C, the relative conversion rate remains at 90.59%, whereas free GDH in the control group has almost lost its activity. The thylakoid-like 3D scaffold matrix of vaterite-Au-EY effectively protects the integrated photosensitive components, ensuring their unique functions in biocatalytic processes under harsh reaction conditions. As shown in Figure 5e, the reusability of vaterite-Au-EY was studied. After the light-driven biocatalytic conversions, vaterite-Au-EY was collected by centrifugation for reuse. It was observed that after three cycles, vaterite-Au-EY still maintained a high relative conversion rate. Compared to previously reported 3D scaffold matrices such as microfluidic chips, peptide nanotubes, and P22 virus-like particles, layered vaterite exhibits excellent low-cost and chemically stable characteristics, capable of withstanding various harsh environments. Moreover, the catalytic reaction’s progress is controllable, as the catalytic process can be directly stopped by centrifugally separating and recovering vaterite-Au-EY. The constructed layered porous structure increases the loading capacity of the photosensitive components, and the interlayer light refraction effectively enhances light absorption [41]. Furthermore, the synergistic surface plasmon resonance of AuNPs aids in enhancing light absorption, further boosting the solar energy capture capability and photoelectron generation in EY. Additionally, compared to traditional spherical vaterite, layered vaterite synthesized using the light-driven method exhibits higher crystalline stability, maintaining its original morphology even after multiple cycles, whereas spherical vaterite is more prone to transformation into calcite.

## 4. Conclusions

Drawing inspiration from the structure and unique function of chloroplast thylakoids in natural photosynthesis, we have fabricated a bio-inspired photosynthesis platform that effectively mimics the unique layered architecture and photoconversion function of thylakoids. By employing layered vaterite as a 3D scaffold to encapsulate the photosensitizer EY and the biocatalyst GDH, and by enhancing light absorption with AuNPs, light-driven NADH regeneration and the sustainable synthesis of L-glutamate have been achieved. The layered structure of the vaterite not only exploits the LSPR effect of loaded AuNPs but also significantly enhances the light absorption of EY through interlayer porous refraction while providing protection for GDH against harsh conditions. Under visible light irradiation, the photocatalytic platform exhibits an initial conversion rate of 0.453 mM/h for the rapid transformation of α-ketoglutarate into L-glutamate. Furthermore, it maintains exceptional stability in adverse environments, displaying excellent relative conversion rates under high pH (pH = 10) or elevated temperatures (37–57 °C).

## Data Availability

Data are contained within the article.
